# Noninvasive characterization of oocyte deformability in microconstrictions

**DOI:** 10.1126/sciadv.adr9869

**Published:** 2025-02-19

**Authors:** Lucie Barbier, Rose Bulteau, Behnam Rezaei, Thomas Panier, Gaëlle Letort, Elsa Labrune, Marie-Hélène Verlhac, Franck Vernerey, Clément Campillo, Marie-Emilie Terret

**Affiliations:** ^1^ Center for Interdisciplinary Research in Biology (CIRB), Collège de France, Université PSL, CNRS, INSERM, 75005 Paris, France.; ^2^ Université Paris-Saclay, Univ Evry, CY Cergy Paris Université, CNRS, LAMBE, 91025 Evry-Courcouronnes, France.; ^3^ Department of Mechanical Engineering, Program of Materials Science and Engineering, University of Colorado, Boulder, CO, USA.; ^4^ Sorbonne Université, CNRS, Institut de Biologie Paris-Seine, Laboratoire Jean Perrin (LJP), Paris, France.; ^5^ Department of Developmental and Stem Cell Biology, Institut Pasteur, CNRS UMR 3738, Université Paris Cité, 25 rue du Dr. Roux, 75015 Paris, France.; ^6^ Hospices Civils de Lyon, Service de médecine de la reproduction et préservation de fertilité, Inserm U1208, SBRI, Faculté de médecine Laennec, Université Claude Bernard Lyon 1, Villeurbanne, France.; ^7^ Institut Universitaire de France (IUF), 75005 Paris, France.

## Abstract

Oocytes naturally present mechanical defects that hinder their development after fertilization. Thus, in the context of assisted reproduction, oocyte selection based on their mechanical properties has great potential to improve the quality of the resulting embryos and the success rate of these procedures. However, using mechanical properties as a quantifiable selective criterion requires robust and nondestructive measurement tools. This study developed a constriction-based microfluidic device that monitors the deformation of mouse oocytes under controlled pressure. The device can distinguish mechanically aberrant oocyte groups from healthy control ones. On the basis of a mathematical model, we propose that deformability measurements infer both oocyte tension and elasticity, elasticity being the most discriminating factor in our geometry. Despite force transmission during oocyte deformation, no long-term damage was observed. This noninvasive characterization of mouse oocyte deformability in microconstrictions allows for a substantial advance in assessing the mechanical properties of mammalian oocytes and has potential application as a quantifiable selective criterion in medically assisted reproduction.

## Teaser

A microfluidic measurement method translates oocyte mechanical characterization into oocyte quality assessment.

## INTRODUCTION

Mechanical parameters have been linked to the biochemical composition and structural organization of cells. Measurement of mechanical properties at the cell level can be performed by imposing a deformation within the micrometer range, using a known force applied to the cell surface. This makes the measurement of mechanical parameters a robust and nondestructive assay that reflects the cell’s physiological or pathological state (*1*). In assisted reproduction, oocyte quality is key for fertilization success and subsequent embryonic development (*2*). However, oocyte morphogenesis is error-prone, and oocytes collected during assisted reproduction are of uneven quality. Preselection of oocytes before fertilization could improve the resulting embryos quality and thus the success rate of these procedures (*3*). Mechanical parameters are among the promising biomarkers of oocyte quality currently under investigation (*4*, *5*), especially as oocytes naturally present mechanical defects that hinder their development after fertilization (*6*).

Compared to somatic cells, oocytes are large spherical cells (80 and 120 μm in diameter for mice and humans, respectively) surrounded by an extracellular matrix layer (called the zona pellucida) only partially connected to the oocyte’s plasma membrane, creating a perivitelline space. Oocytes ready for fertilization are arrested in metaphase of second meiotic division (meiosis II) with their chromosomes aligned on the microtubule spindle. The mechanical properties of oocytes and of the zona pellucida have been extensively studied over the last decade. The zona pellucida has been characterized as a compressible elastic material by micro and nano-indentation experiments (*7*, *8*). Using micropipette aspiration after removal of the zona pellucida, it was shown that oocyte surface tension is controlled by cortical myosin-II localization and actin nucleation beneath the plasma membrane, closely related to oocyte developmental stage (*9*–*11*). Several studies have focused on assessing the subcellular mechanical properties of oocytes (*12*, *13*), and probing of the cytoplasm with optical tweezers reveals homogeneous viscoelastic properties (*14*). The overall deformability of oocytes with their zona pellucida under compression correlates with morphological quality criteria (*15*, *16*), and bulk viscoelastic measurements using micropipette aspiration can be used to differentiate between viable and nonviable one-cell embryos (*6*, *17*). In addition, the elasticity of the zona pellucida and the viscosity of the cytoplasm are correlated with the quality of human oocytes (*18*–*20*). Furthermore, cortical tension defects affect chromosome segregation and oocyte division geometry, which have an impact on postfertilization development (*9*, *11*, *21*). Overall, this body of work suggests that oocyte mechanical properties have a great potential as quantifiable biomarkers of oocyte quality. However, their use as a selective criterion for assisted reproduction raises the need for adapted measurement tools, and only few studies focus on a minimally invasive methodology using a platform transferable to the clinical environment (*12*, *15*, *19*, *20*, *22*).

Recent works have focused on the development of microfluidic-based methods enabling application of cells mechanical measurements as biomarkers for medical diagnostics. Microfluidic devices feature structures with characteristic lengths similar to those of cells, and objects can undergo controlled deformations on the micrometer scale in well-defined geometries and flows. Moreover, they are easy to use and their prototyping is versatile and inexpensive, which favors the development of new tools for medical diagnostic purposes (*23*, *24*). Over the past decade, microfluidic devices processing blood samples through constrictions smaller than typical cell size have been developed for the medical detection of mechanically aberrant red blood cells, as in case of malaria infection (*25*, *26*) or of tumor circulating cells (*27*, *28*). In constriction-based methods, cell deformability is inferred by measuring the entry time, elongation, and/or the pressure required for cell passage (*28*, *29*). Quantitative studies have applied rheological models, such as a power law to determine single-cell elastic modulus and apparent viscosity (*30*–*32*), or Laplace’s law to determine cell surface tension (*33*). Constriction-based microfluidic devices could represent a promising method for assessing the mechanical properties of oocytes for medical applications. To the best of our knowledge, only two studies have proposed the measurement of oocyte deformability using microconstrictions (*34*, *35*). However, they both reported oocyte damage due to flow-induced shear stress during transit through the constriction (*34*).

Here, we designed a square microconstriction with a smaller cross section but shorter length than in previous studies. In this configuration, mouse oocytes completely fill the narrowed channel and are therefore not subjected to shear stress induced by fluid flow. We were able to measure the pressure required for oocyte passage, while assessing global and subcellular deformation. By comparing groups with known mechanical properties, we were able to distinguish mechanically aberrant oocytes from healthy control ones. On the basis of a mathematical model, we propose that our deformability measurements infer both oocyte cortical tension and elasticity. At the subcellular level, we were able to assess meiotic spindle deformation in the constriction. After recovery, no morphological features could distinguish oocytes that passed the constriction with the off-chip control groups. Thus, we demonstrate that under reduced shear conditions, characterization of mouse oocyte deformability in microconstrictions can be noninvasive. Our study focuses on the measurement and modeling of global deformation and opens the possibility of a more precise analysis of the subcellular mechanical properties of oocytes in our microconstrictions.

## RESULTS

### Principle of the constriction-based deformability assay

**Fig. 1.**
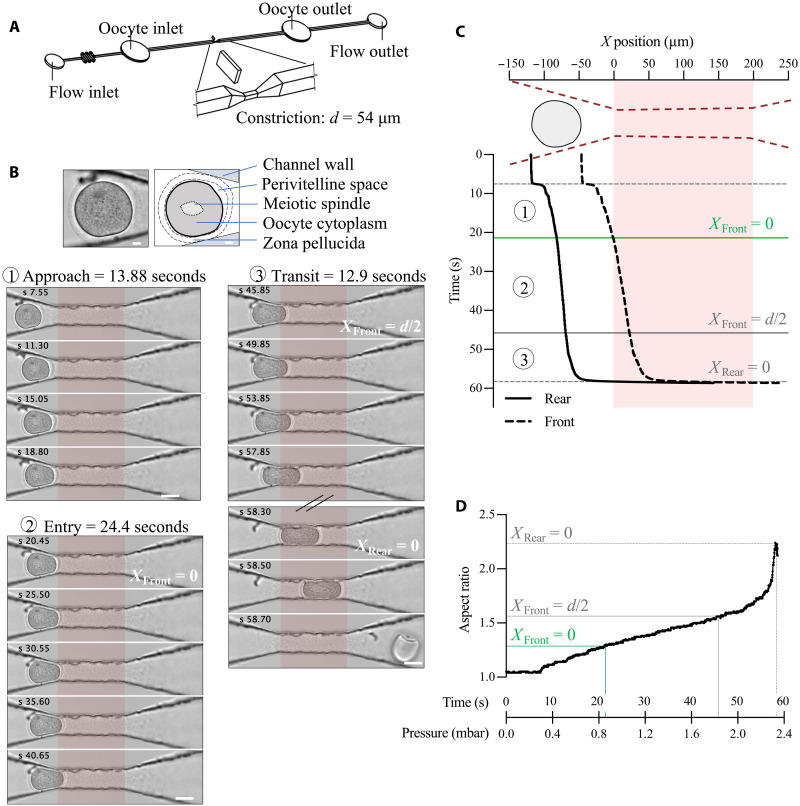
Constriction-based deformability assay for mouse oocytes. (**A**) 3D design of the microfluidic device. The zoom shows the 54-μm square constriction in the center of the channel. (**B**) Image sequence of a representative passage of an oocyte through the constriction. Upper panel: image and annotated diagram of the oocyte before entering the constriction. Scale bar, 10 μm. The bottom panel shows the three phases of deformation we have identified: approach (1), entry (2), and transit (3). The first image of the sequence corresponds to zero flow in the channel (see fig. S1D). The images for which the front of the oocyte enters the constriction (*X*_Front_ = 0), the front of the oocyte reaches half the smallest dimension of the constriction (*X*_Front_ = *d*/2), and the rear of the oocyte enters the constriction (*X*_Rear_ = 0) are annotated. Scale bar, 50 μm; time in seconds and constriction highlighted in red. (**C**) Front (dotted line) and rear (solid line) position of the oocyte shown in (B) relative to the start of the constriction. The drawing shows the constriction in dotted red and the initial oocyte position in gray. The constriction is highlighted in red throughout the graph. The first horizontal dotted line indicates the time of zero flow in the channel. The following lines correspond to the annotated images in (B) *X*_Front_ = 0, *X*_Front_ = *d*/2, and *X*_Rear_ = 0. (**D**) Aspect ratio of the oocyte shown in (B) as a function of time (in seconds) since the start of the assay and corresponding applied pressure (in mbar). The lines show the aspect ratio and pressure for the three annotated images in (B) *X*_Front_ = 0, *X*_Front_ = *d*/2, and *X*_Rear_ = 0.

In this work, we study oocyte deformation under precisely controlled pressure. To this end, we designed a constriction in a microfluidic channel that restricts the size of the oocyte in two dimensions (Fig. 1A). The restriction corresponds to a 200-μm-long segment over which the channel cross section remains almost square but is reduced to a minimum size of 54 × 62 μm. Two 300-μm-long ramped segments provide a size transition between the main channels and the constriction (fig. S1A). For deformation measurement, a single mouse oocyte is brought to the ramp segment by a moderate flow (2 μl/min). The flow is then reduced to 0 and the inlet pressure increased by 0.1 mbar every 2.5 s until the oocyte passes through the constriction (Fig. 1B and fig. S1C). Once trapped in the constriction, the oocyte blocks the flow (fig. S1D), so the applied pressure is equivalent to the inlet pressure. Moreover, oocyte elongation is only possible along the channel direction, and can therefore be measured using single-*z* imaging (Fig. 1B and movie S1).

We used the Oocytor plugin (*36*) to extract the contour of the oocytes, excluding their zona pellucida, from bright-field images of their passage through the constriction. We thus obtained the front and rear positions of oocytes with respect to the constriction entry (denoted by *X* = 0 in Fig. 1C, the constriction is highlighted in red as in Fig. 1B) as well as their aspect ratio (Fig. 1D) as a function of the applied pressure. We identified three phases in oocyte deformation (Fig. 1, B and C): (i) the approach phase, that starts when triggering the stepwise pressure increase and ends when the front of the oocyte enters the constriction (at which we defined the pressure *P*_*X*f=0_). At this point, the oocytes completely block the flow under all the conditions tested; (ii) the entry phase, during which the rear of the oocyte remains quasi-static while the front advances into the constriction. This phase is characterized by a linear increase in the oocyte’s aspect ratio, and we can extract the pressure at which the front of the oocyte reaches half the smallest dimension of the constriction (*P*_*X*f=*d*/2_); and (iii) the transit phase, which corresponds to a clear displacement of the oocyte’s rear and is characterized by a rapid, nonlinear increase in its aspect ratio. During this phase, we can extract the pressure at which the oocyte is completely deformed in the constriction (*P*_*X*r=0_) (Fig. 1, B to D).

We have thus built a tool able to monitor in-live mouse oocyte deformation as a function of applied pressure and identified three regimes associated with critical pressure values designated as *P*_*X*f=0_, *P*_*X*f=*d*/2_, and *P*_*X*r=0_.

### Measurement of deformability identifies oocytes with aberrant mechanical properties

**Fig. 2.**
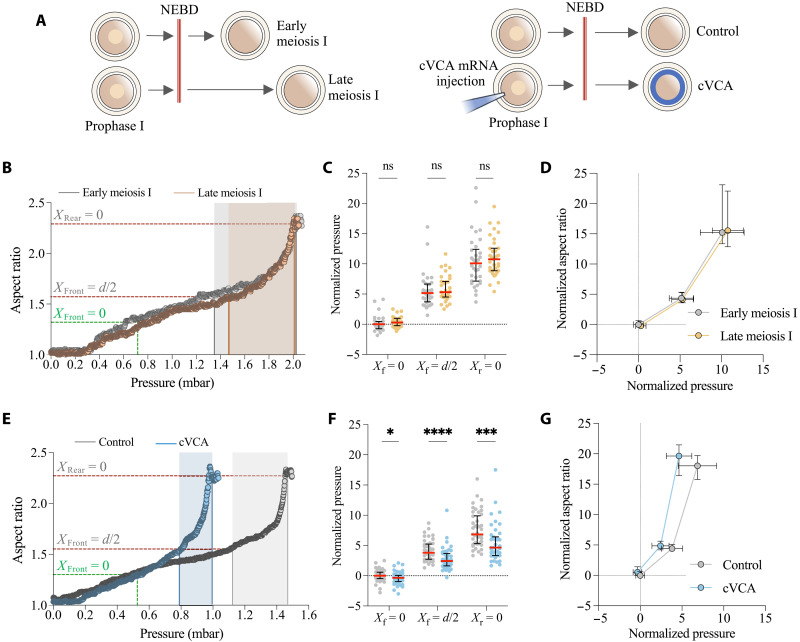
Measurement of pressure and aspect ratio during transit through the constriction for mechanically distinct groups of oocytes. (**A**) Diagram of methods to obtain the four oocyte groups from oocytes arrested in prophase I. The double red bar indicates the resumption of meiotic division identified by NEBD. Early meiosis I corresponds to oocytes 2 to 3 hours after NEBD; late meiosis I to oocytes 6 to 8 hours after NEBD. cVCA oocytes are obtained by microinjection of cVCA mRNA in prophase I, and measured 4 hours after NEBD. (**B**) Oocyte aspect ratio as a function of applied pressure for a representative early meiosis I oocyte (gray) and a representative late meiosis I oocyte (orange). The lines show the aspect ratio and pressure (in mbar) for the three critical points: *X*_Front_ = 0 (*X*_f_ = 0), *X*_Front_ = *d*/2 (*X*_f_ = *d*/2), and *X*_Rear_ = 0 (*X*_r_ = 0) as described in Fig. 1. The filled-in area highlights the pressure difference between *X*_f_ = *d*/2 and *X*_r_ = 0. (**C**) Normalized pressure measured at the three critical points for early and late meiosis I oocytes and (**D**) median of normalized oocyte aspect ratio as a function of the median of the applied pressure for *X*_f_ = 0, *X*_f_ = *d*/2, and *X*_r_ = 0. *n* = 34 for early meiosis I and *n* = 35 for late meiosis I from two independent experiments. For each experiment, values are normalized to the median and interquartile range obtained for early meiosis I at *X*_f_ = 0. (**E** to **G**) Same as (B) to (D), respectively, for control (gray) and cVCA oocytes (blue). *n* = 46 for control and *n* = 43 for cVCA oocytes from four independent experiments. For each experiment, values are normalized to median and interquartile range obtained for control oocytes at *X*_f_ = 0. Error bars show median and interquartile range; ns, *P* > 0.05; *P = 0.0244, ***P = 0.0001, and ****P < 0.0001 to Mann-Whitney statistical test.

We hypothesized that oocyte mechanical parameters could be inferred from the measurement of pressure and aspect ratio during transit through the constriction. To test this hypothesis and identify the most discriminating parameters, we compared groups of mouse oocytes with known different mechanical properties (Fig. 2A). First, we tested healthy control oocytes at early and late stages of their first meiotic division (meiosis I), since cortical tension decreases as meiosis I progresses (Fig. 2A, left diagram) (*9*, *10*). We found no significant difference either in the pressure required for oocyte transit or in the aspect ratio inside the constriction between these two groups (Fig. 2, B to D; fig. S2; and movie S2). Second, we used a model of extra-soft oocytes microinjected before meiosis I, in prophase I, with cRNA encoding cortical verprolin, cofilin, acidic (VCA) (cVCA oocytes), resulting in ectopic cortical actin polymerization via the Arp2/3 complex and myosin-II chasing from the cortex. This modification of cortical actomyosin organization induces aberrant mechanical properties, cVCA oocytes having a lower cortical tension compared to control oocytes, leading to aberrant phenotypes (Fig. 2A, right diagram) (*9*, *11*, *21*). In our assay, the pressure required for cVCA oocytes to deform through the constriction (*P*_*X*r=0_) was significantly lower than for the control oocytes, while the oocyte aspect ratio remained similar. The pressures *P*_*X*f=0_ and *P*_*X*f=*d*/2_ were also significantly lower for cVCA than for control oocytes (Fig. 2, E to G; fig. S2; and movie S3).

In parallel, we tested the performance of oocyte discrimination by visual inspection of transmitted light images, as done in medically assisted reproduction, and increasingly supported by artificial intelligence (AI)–based image analysis. We used Oocytor, an AI-based image analysis pipeline that we use routinely (*36*–*38*), to automatically extract and compare morphological features of control and cVCA oocytes before entering the constriction. We trained a random forest classifier and estimated its performance to discriminate between oocyte types with a cross-validation scheme (see Materials and Methods). We obtained a classification accuracy of 57% (with a precision of 56% and a recall of 64%). An accuracy of 57% is barely better than a random chance (50%) and suggests a limited discriminating capacity. Thus, and contrary to the device, the AI approach failed to identify oocytes with aberrant mechanical properties.

Measurements of the pressure required to pass through the constriction distinguishes oocytes with aberrant mechanical properties, but not healthy control oocytes at different stages of meiosis I. Measurement of the maximal oocyte aspect ratio was similar in all the tested conditions (fig. S2B). For early versus late meiosis I and for cVCA versus control oocytes, differences in oocyte cortical tension have been established using micropipette aspiration (*9*, *10*). However, other mechanical properties such as oocyte elasticity and viscosity have not been well characterized and could explain the difference in the ability to distinguish these groups of oocytes. In particular, for droplet models used to analyze micropipette aspiration experiments, surface tension governs deformability and the threshold pressure required for droplet entry in the micropipette is reached when the droplet front in the micropipette is at half the pore size (*X*_f_ = _*d*/2_) (*39*). However, in our experiment, *P*_*X*r=0_ was significantly higher than *P*_*X*f=*d*/2_ in all the conditions tested (Fig. 2, D and G). This suggests that cortical tension is not the only mechanical parameter governing oocyte deformability through the constriction. To further characterize the effect of variations in tension and elasticity on the deformability of the oocyte through the constriction, we turned to mathematical modeling.

### Tensile elastic shell modeling highlights elasticity as the determining factor in object deformation

We constructed a simplified mathematical model of the oocyte based on a classical droplet model, combined with an elastic component to represent the solid-like response of the cortex (*40*). The oocyte is modeled as a spherical shell with both surface tension and elastic properties. Thus, the surface stress τ can be written as follows

$$\tau =\tau_{0}+E\times t_{h}\left(\frac{A}{A_{0}}-1\right)$$ (1)

**Fig. 3.**
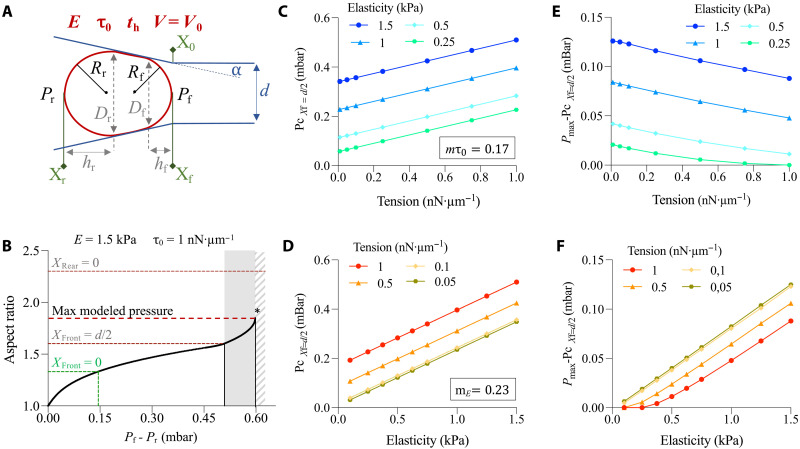
Modeling the deformation of a tensile elastic shell through the constriction. (**A**) Diagram of the parameters used in the mathematical model. Shell parameters are shown in red: surface tension $\tau_{0}$, elastic modulus *E*, mean shell thickness $t_{h}$, volume conservation *V* = *V*_0_. The geometric parameters of the constriction are in blue: minimal diameter *d* and inclination of the ramped segment α. Oocyte front (*X*_f_) and rear (*X*_r_) positions relative to the constriction entry (*X*_0_) are in green. In black: *R*_f_ and *R*_r_ and *P*_f_ and *P*_r_ are the radii and pressures of the front and rear edges used to apply the Laplace law. In gray: $h_{f}$ and $h_{r}$ and $D_{f}\;\text{and}\;D_{r}$ are the heights of the front and rear caps and the channel diameters at the base of the front and rear caps used to calculate the equilibrium pressure difference associated with a given cell configuration (Materials and Methods). (**B**) Evolution of shell aspect ratio as a function of pressure gradient $P_{f}-P_{r}$ calculated with *D*_0_ = 74.4 μm, *E* = 1.5 kPa, $\tau_{0}$=1 nN·μm^−1^, α = 9°, *d* = 50 μm, and *t*_h_ = 24.4 μm. The lines indicate the aspect ratio and pressure for the three critical points: *X*_Front_ = 0, *X*_Front_ = *d*/2, and the maximum pressure *P*_max_ reached with the modeling. The gray area indicates the pressure difference between *P*_*X*f=*d*/2_ and *P*_max_. *X*_Rear_ = 0 corresponds to a hypothetical line and the striped area indicates the deformations observed in our experimental setup but not reached by the model. (**C**) Critical pressure at *X*_f_ = *d*/2 and (**E**) pressure difference between *P*_*X*f=*d*/2_ and *P*_max_ as a function of shell tension calculated for different elasticity values. *m*$\tau_{0}$ indicate the slope of the linear regression for the four elasticity conditions in (C). (**D**) Critical pressure at *X*_f_ = *d*/2 and (**F**) pressure difference between *P*_*X*f=*d*/2_ and *P*_max_ as a function of shell elasticity calculated for different tension values. *m_E_* indicates the slope of the linear regression calculated for the four tension conditions in (D).

With $\tau_{0}$ the surface tension, *E* the elastic modulus, $t_{h}$ the mean shell thickness, while *A* and *A*_0_ are the current and stress-free reference surface area of the shell, respectively. We can further idealize the oocyte shape as an axisymmetric conical geometry capped with two hemispherical shells as depicted in Fig. 3A. In these conditions, we can use the Laplace’s law to obtain the following relationship between internal shell pressure *P*_in_ and the pressures *P*_f_ and *P*_r_ applied at the front and rear edges, respectively

$$P_{\text{in}}-P_{f}=2\frac{\tau }{R_{f}}\;\text{and}\;P_{\text{in}}-P_{r}=2\frac{\tau }{R_{r}}$$ (2)

where *R*_f_ and *R*_r_ are the radii of the front and rear edges, respectively (Fig. 3A). Furthermore, considering the pressure applied to the whole shell, we combine equations in Eq. 2

$$P_{r}-P_{f}=2\tau \left(\frac{1}{R_{f}}-\frac{1}{R_{r}}\right)$$ (3)

Using Eq. 1, Eq. 3, and conservation of oocyte volume (*7*), we can calculate the equilibrium pressure difference $P_{f}-P_{r}$ associated with a given cell configuration (see Materials and Methods). As our experimental measurement of oocyte contours excludes the zona pellucida, we corrected the model’s geometric parameters, *d* the constriction width, and α the inclination of the ramped segment (Fig. 3A), to obtain aspect ratio values similar to the experimental data without accounting for zona pellucida thickness (fig. S3A). With values in the range of previous publication, *E* = 1.5 kPa and $\tau_{0}$=1 nN·μm^−1^ (*8*–*10*, *12*), the evolution of the shell aspect ratio as a function of the pressure difference $P_{f}-P_{r}$ follows a similar trend to that observed in our experiments (Fig. 3B). The mathematical model shows a pressure threshold *P*_max_ beyond which the passage of the shell through the constriction does not require any pressure increase. For this critical pressure, the shell is not yet fully deformed inside the constriction, so the pressure *P*_*X*r=0_ observed in our experimental setup is not reached by the model.

If we applied Laplace’s law and considered only surface tension, the threshold pressure *P*_max_ would be reached when the front radius is equal to *d*/2 (*P*_*X*f=*d*/2_). In the case of our model including an elastic component, the threshold pressure *P*_max_ is higher than *P*_*X*f=*d*/2_ (Fig. 3B). Using this mathematical model, we could assess the effect of varying tension and/or elasticity on *P*_*X*f=*d*/2_ and *P*_max_. We found that *P*_*X*f=*d*/2_ follows a positive linear relationship with tension or elasticity, the slope being higher with variations in the shell elasticity than shell tension (Fig. 3, C and D). We calculated the analytical expressions explaining the linear relationship between *P*_*X*f=*d*/2_ and shell tension (slope, *m*$\tau_{0}$) or elasticity (slope, *m_E_*) as a function of our model geometry (see Materials and Methods). Decreasing constriction width *d* increases both *m*$\tau_{0}$ and *m_E_*, while increasing ramp inclination α between 10° and 50° favors *m*$\tau_{0}$ over *m_E_* (fig. S3, B and C). Last, the difference between *P*_max_ and *P*_*X*f=*d*/2_ depends mainly on the elasticity of the shell and decreases only slightly with tension. As expected, *P*_max_-*P*_*X*f=*d*/2_ tends toward 0 when elasticity decreased (Fig. 3, E and F). The results are similar if we consider a more realistic pyramidal shape instead of an axisymmetric conical geometry (fig. S3, D and E).

Overall, *P*_*X*f=*d*/2_ reflects both shell tension and elasticity. In our geometry, *P*_*X*f=*d*/2_ is more influenced by variations in elasticity than in tension. Moreover, the difference between *P*_max_ and *P*_*X*f=*d*/2_ mainly reflects a variation in elasticity. A critical pressure corresponding to the modeled *P*_max_ is difficult to extract from our experimental measurements. However, if we approximate this threshold value by the pressure *P*_*X*r=0_, we find that the difference between *P*_*X*r=0_ and *P*_*X*f=*d*/2_ is smaller in cVCA oocytes than in control oocytes (fig. S3F), suggesting that cVCA oocytes have lower elasticity than control oocytes. These changes in elasticity could explain the differences in pressure measured between cVCA and control oocytes.

Together, our tool is able to distinguish mechanically aberrant oocytes from healthy control ones, with deformability measurements inferring both oocyte tension and elasticity, elasticity being the most discriminating factor in our geometry. For medical use, our tool must be noninvasive, which we then tested.

### Meiotic spindles recover normal morphologies within hours after oocyte measurement

**Fig. 4.**
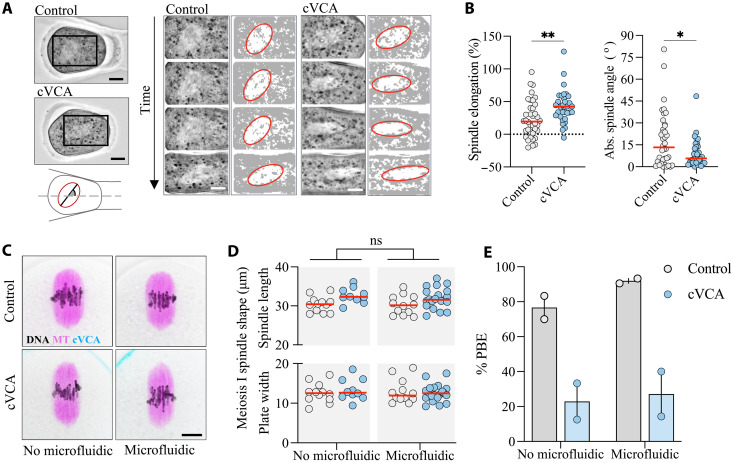
Meiotic spindle deformation and recovery after passage through constrictions. (**A**) Control and cVCA oocytes approaching the constriction with insets encompassing their spindle; scale bars, 10 μm. The diagram illustrates measurements in (**B**) of spindle major axis and angle to channel axis. The sequences of images cropped from the left oocytes show an image every 15 (control) and 10 s (cVCA). Binarized images with the spindle contour in red are shown. Scale bars, 15 μm. Left: Percentage of spindle elongation relative to its length before oocyte deformation in the constriction. Right: Absolute value of the spindle long axis angle relative to the direction of the channel measured once the oocyte is fully deformed in the constriction (i.e., *X*_Rear_ = 0). *n* = 38 control and *n* = 33 cVCA from four independent experiments. **P = 0.0038 by *t* test; *P = 0.0388 by Kolmogorov-Smirnov test. (**C**) Spindles of control and cVCA oocytes 2 hours after passage through the device (right) or unmanipulated (left). Microtubules are in magenta, DNA in purple, cVCA in cyan, and bright-field images in gray. Scale bar, 10 μm. (**D**) Spindle length and metaphase plate width for control and cVCA oocytes measured from (C). No microfluidic: *n* = 12 control and *n* = 10 cVCA; microfluidic: *n* = 12 control and *n* = 20 cVCA from three independent experiments. ns, *P* > 0.05 for no-microfluidic versus microfluidic by two-way analysis of variance (ANOVA) test applied to spindle length and plate width. For all graphs, red bars represent the median. (**E**) Percentage of control and cVCA oocytes extruding a polar body (PBE) after recovery from the device or unmanipulated. No microfluidic: *n* = 22 control and n = 14 cVCA; microfluidic: *n* = 35 control and *n* = 25 cVCA from two independent experiments (represented by a dot). The bars represent the median.

One of the main limitations of using constrictions to assess oocyte deformability is the shear stress–induced damage to the meiotic spindle described in previous work (*34*), critical for oocyte viability. To investigate the impact of our assay on the meiotic spindle, we first monitored whether it was subjected to forces during transit through the constriction in meiosis I. Spindles are visible in bright-field images as a more light-transparent area, excluding dark cytoplasmic granules. We were able to manually track the spindles during the assay, analyzing their elongation and rotation (Fig. 4A). We found that the spindles were indeed subjected to forces as they passed through the constriction, elongating by 25% along their long axis and tending to align with the channel axis (Fig. 4B and fig. S4A). Thus, during oocyte transit through the constriction, forces are transmitted through the cytoplasm, inducing spindle deformation. The spindles of cVCA oocytes elongate more and are more aligned with the channel axis than those of control oocytes (Fig. 4B and fig. S4A), suggesting an alteration in the cytoplasmic mechanical properties of cVCA oocytes, reinforcing the conclusion that our deformability assay assesses more than just differences in cortical tension. To test whether passage through the constriction induces long-term alteration of the meiotic spindle, we retrieved oocytes from the microfluidic device and assessed spindle length and chromosome alignment on the metaphase plate 2 hours after deformation (Fig. 4C). We found no morphological difference between meiotic spindles of control or cVCA oocytes passed through the microfluidic constriction and their unmanipulated counterparts (Fig. 4D). Last, control and cVCA oocytes completed meiosis I, marked by polar body extrusion (PBE), at the same rate as their unmanipulated counterparts (Fig. 4E). Overall, despite transient spindle deformation during the passage through the constriction, we did not identify any long-term impact, oocytes reaching meiosis II with no visible morphological alteration.

**Fig. 5.**
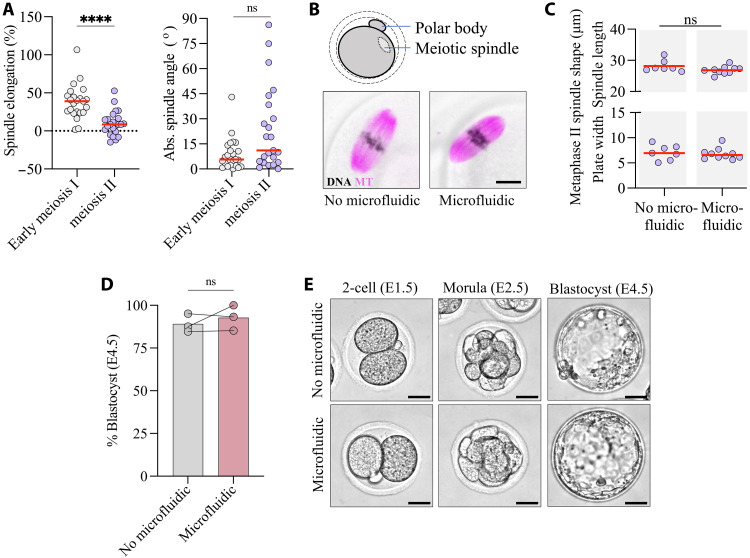
Recovery of oocytes in meiosis II and zygotes after passage through constrictions. (**A**) Left: Percentage of spindle elongation relative to its length before oocyte deformation in the constriction. Right: Absolute value of the spindle long axis angle relative to the direction of the channel measured once the oocyte is fully deformed in the constriction (i.e., *X*_Rear_ = 0). *n* = 23 oocytes in early meiosis I and *n* = 23 oocytes in meiosis II from three independent experiments. ****P < 0.0001 by *t* test; ns, *P* = 0.0591 by Kolmogorov-Smirnov test. (**B**) Spindles of oocytes in meiosis II 2 hours after passage through the device (right) or unmanipulated (left). Microtubules are in magenta, DNA in purple, cVCA in cyan, and bright-field images in gray. Scale bar, 10 μm. The diagram shows an oocyte in meiosis II with an extruded polar body and a spindle located beneath the cell cortex. (**C**) Spindle length and metaphase plate width for oocytes in meiosis II measured from (B). *n* = 7 for no microfluidic and *n* = 10 for microfluidic from one experiment. ns, *P* > 0.05 by Mann-Whitney test applied to spindle length and plate width. For all graphs, red bars represent the median. (**D**) Percentage of zygotes developing up to the blastocyst stage after recovery from the device, or unmanipulated. No microfluidic: *n* = 83, microfluidic: *n* = 87 from three independent experiments (two female mice per experiment) represented by a dot. The bars represent the median. (**E**) Embryos at different stages of development coming from zygotes recovered from the device (bottom) or unmanipulated (top). Scale bars, 30 μm.

In the standard protocols for medically assisted reproduction, hormonal treatments induce the resumption of the first meiotic division in the ovary, and oocytes that are retrieved from punctures are in meiosis II. At that stage, they are arrested in metaphase until fertilization by a sperm. Oocytes arrested in meiosis II have completed meiosis I, extruded a first polar body, and their spindle is located beneath the cell cortex, anchored in an actomyosin network. To bring our research closer to the medical application, we tested whether our microfluidic device could allow to measure and recover meiosis II oocytes without causing damage, in particular to their meiotic spindle. During transit through the constriction, the spindle of meiosis II oocytes appeared to be subjected to fewer forces than in early meiosis I. This was evidenced by the spindle being less elongated and showing a reduced tendency to align along the channel axis (Fig. 5A, fig. S4B, and movie S5). After recovery, we found no morphological alterations in meiosis II oocytes, with no differences in spindle length nor chromosome alignment in meiosis II between those manipulated in the microfluidic devices and those that were not (Fig. 5, B and C).

Last, the gold standard for testing the safety of materials and media used in human medically assisted reproduction is the mouse embryo assay, which evaluates embryonic development up to the blastocyst stage after exposure to the tested material (*41*). We thus tested the development to blastocyst stage of mouse zygotes after passage through the device (movie S6). The zygotes that passed in the device developed up to the blastocyst stage at the same rate as their unmanipulated counterparts (Fig. 5, D and E). Furthermore, we found no morphological differences between blastocysts derived from zygotes passed through the microfluidic constriction and their unmanipulated counterparts (Fig. 5E and fig. S4C). Together, our results suggest that characterizing oocyte deformability in our constrictions is noninvasive, indicating potential clinical applications.

## DISCUSSION

In this work, we describe a deformability assay using a constriction in a microfluidic device, which enables the monitoring of oocyte deformation as a function of a known applied pressure. By measuring the pressure required to pass through the constriction, we can distinguish oocytes with aberrant mechanical properties from healthy control ones, but not healthy control oocytes at different stages of meiosis I. On the basis of a mathematical model combining tension and elasticity, we propose that the elastic parameter primarily governs oocyte deformability through constriction, over surface tension. At the subcellular level, we show that the meiotic spindle deforms in the constriction. However, after recovery, no morphological features distinguish oocytes that passed the constriction from off-chip control groups, suggesting that the deformability assay may be noninvasive and could be used for clinical applications.

Recent works to assess the mechanical properties of oocytes have focused on small deformations using an indenter or aspiration pipette derived from intracytoplasmic sperm injection protocols (*17*, *20*). However, small deformations only probe the mechanical properties of the zona pellucida and do not allow to infer the overall mechanical properties of the oocyte. On the contrary, microfluidic constrictions induce large deformations, probing the mechanical properties of the oocyte, and can easily be integrated into an on-chip platform (*42*). However, the only two studies that measured oocyte deformability using microconstrictions have raised concerns about their impact on oocyte viability (*34*, *35*). Luo *et al.* (*34*) designed a constriction of 50 μm wide and 150 μm high by 200 μm long and evaluated oocyte entry time under relatively high flow rates (e.g., 10 and 20 μl/min). Saffari *et al.* (*35*) determined the cortical tension of oocytes by measuring the pressure required for their passage through a constriction of 75 μm wide and 125 μm high by 700 μm long. Both studies were carried out on a small sample size (fewer than 30 oocytes) and reported oocyte damage: Only 13 of the 23 tested oocytes reached full maturation in Saffari *et al.*, while Luo *et al.* described shear stress–induced spindle damage in 59% of tested oocytes, compared with 20% in the off-chip control groups. Our results demonstrate that constriction geometry is critical for oocyte viability. Compared to previous works, we used a constriction with a smaller cross section but shorter length, limiting shear stress induced by fluid flow around the oocyte. As a result, we found no morphological signs of oocyte damage, and zygotes that passed through the constriction developed up to the blastocyst stage in vitro. Further viability tests in mice, such as reimplantation into female recipient mice, are necessary to confirm developmental potential after deformation. For use in clinical applications, the assay should be further adapted for use with human oocytes, and rigorously tested to ensure it is noninvasive and compatible with oocyte and embryo development. In medically assisted reproduction, oocyte quality assessment is based on morphological criteria, recently supported by AI-based image analysis (*3*). However, and in contrast to the device, this approach failed to identify oocytes with aberrant mechanical properties, showing that the information provided by passage pressure is not redundant with visual inspection. In a broader context, the measurement of mechanical properties as proposed in this work could be combined with AI-based morphological analysis to better identify high-quality oocytes.

In previous constriction-based methods, cell deformability was described either using a power law rheological model describing viscoelastic cell behavior (*30*–*32*), or by considering cells as Newtonian liquid droplets and applying the Laplace’s law to access surface tension properties (*33*). Dupire *et al.* (*43*) considered cells as viscoelastic droplets with surface tension generated by the actin cortex. In their model of the flowing cell, the cortical tension limits the effective stress applied to the viscoelastic material. In our model, we consider oocytes as incompressible spherical shells with surface tension and elastic properties, but we do not include bulk viscous properties. This minimal model was sufficient to account for oocyte deformation through constrictions. Using this modeling approach, we found that the constriction geometry is crucial to measurement sensitivity and accuracy. The angle α of the ramp section determines the mechanical parameter that governs the oocyte entry pressure: An angle α between 10° and 50° favors probing of cortical tension, while a higher or lower angle α favors probing of elasticity (fig. S3C). Our results show that a range of mechanical parameters can be inferred in the same deformability assay with appropriate constriction geometries. This may enable to study the interplay between several mechanical parameters during oocyte morphogenesis and thus assess which parameter(s) is/are the most effective for inferring oocyte quality. In addition, we found that increasing the initial shell size (*D*_0_) while maintaining the same ratio of size to constriction width (*D*_0_/*d*) results in a decrease in measurement accuracy for cortical tension but not for elasticity (fig. S3, G and H). This result is essential for adapting the microconstriction method to the measurement of larger oocytes from other species, such as human oocytes, which have a median diameter of 114.7 μm compared to 73.09 μm for mouse oocytes, but with similar relative size variation in the population (see Materials and Methods). Thus, the shell model could be used to determine an optimal geometry that minimizes oocyte deformation while ensuring accurate measurement for oocytes selection before in vitro fertilization. To further complete these results, additional elements could be implemented in the mathematical model, including oocyte viscosity, characteristics of the zona pellucida, perivitelline space, and the presence of a polar body in meiosis II oocytes, as these factors might constrain oocyte deformation and influence measurement accuracy.

Last, one of the strengths of our approach is to combine the measurement of force and deformation with high-quality live imaging. Without specific staining, we are able to segment subcellular elements of the oocyte as it deforms through the constriction. We present manual quantification of meiotic spindle deformation, but we were also able to observe cytoplasmic flows and thinning of the zona pellucida. Further developments in image processing and analysis would be required to quantify these observations. In particular, taking into account the three-dimensional (3D) volume of the oocyte and displacement out of the focal plane would be crucial to the accuracy of these quantifications. Nevertheless, our image data analysis adds valuable information for studying load-transfer mechanisms inside the oocyte, as well as for decoupling the mechanical responses of cellular components (*44*). From a physical standpoint, this would allow to better describe the material properties of oocyte components. From a cell biology perspective, it would enable further studies of the cellular response induced by the mechanical load to which the oocyte may be subjected routinely in clinics during the in vitro fertilization procedures.

## MATERIALS AND METHODS

### Mold microfabrication and scanning electron microscopy imaging

The microfluidic chip was designed on Clewin 5.4 (WieWeb software). The corresponding chromium mask was fabricated with the μPG 101 maskless aligner (Heidelberg Instruments Mikrotechnik GmbH) and the final silicon mold with the MJB4 alignment system (Karl Süss). To obtain 200-μm-thick channels, two 100-μm-thick photoresist dry films were successively laminated onto the silicon wafer. In a second step, the excess height of the photoresist at the constriction was removed using a computer numerical control (CNC) micro-milling machine (Minitech, Machinery Corp). To prevent the polymer sticking to the structures, the wafer was coated with a fluorinated silane by vapor deposition (448931-Trichloro 1*H*,1*H*,2*H*,2*H*-perfluorooctyl silane, Merck-Sigma-Aldrich). For scanning electron microscopy, a 10-nm-thick layer of gold was deposited using sputtering techniques to make the micromilled constriction reflective to electrons. The surface morphology of the constriction was imaged by cold field–emission scanning electron microscopy at an operating voltage of 10 kV and an angle of 35°.

### Microfluidic device fabrication

Liquid polydimethylsiloxane (PDMS; RTV 615, Momentive Performance) in a 1:10 base/cross-linker ratio was poured onto the custom mold, degassed, and cured for at least 2 hours at 70°C. After cutting the PDMS pieces and punching the inlets and outlets with a biopsy punch (Electron Microscopy Sciences, Hatfield), the PDMS pieces were cleaned by sonication in isopropanol for 30 s and left to dry overnight. The PDMS pieces were then bonded to a microscopy glass slide after O_2_ plasma treatment of both surfaces (50 W for 1 min, O_2_ flow rate of 20 sccm, and pressure of 0.15 torr; Cute plasma oven, Femto Science), and left for 5 min at 90°C to ensure bonding. The microfluidic devices produced were connected to a pressure controller and flow sensor (Fluidgent), filled with culture medium and left for at least 1 hour to equilibrate at 37°C before measuring oocytes.

### Mouse oocytes collection and culture

Oocytes were extracted by shredding ovaries collected from 8- to 11-week-old OF1 female mice (Charles River Laboratories) in homemade M2 medium (*45*) supplemented with 1 μM milrinone (*46*) to synchronize them in prophase I. Meiosis resumption is triggered by transferring oocytes into milrinone-free homemade M2 medium.

All live culture and imaging were carried at 37°C and under oil apart for microfluidic measurement. All animal studies were performed in accordance with the guidelines of the European Community and were approved by the French Ministry of Agriculture (authorization D750512).

### Mouse zygote collection, passage through the constriction, and culture

Zygotes were isolated from 8-week-old superovulated OF1 females mated with OF1 males. Superovulation of female mice was induced by intraperitoneal injection of 5 IU of pregnant mare’s serum gonadotropin (Syncro-part, Ceva), followed 48 hours later by intraperitoneal injection of 5 IU of human chorionic gonadotropin (Chorulon, MSD Animal Health). Zygotes were recovered at embryonic day 0.5 (E0.5) by opening the ampulla, followed by a brief treatment at 37°C with hyaluronidase (0.3 mg/ml; H4272-30MG; Sigma-Aldrich) and washing in M2 medium at 37°C. Half of the zygotes were passed through the microfluidic device. After deposition in the inlet, the zygotes were brought to the constriction by a moderate flow and the inlet pressure was increased by 0.2 mbar every 2.5 s until all the zygotes passed through the constriction. For couples of zygotes, images were taken every 50 ms on an inverted bright-field microscope (Diaphot 300, Nikon) with a 20× dry objective and complementary metal-oxide semiconductor (CMOS) camera (ORCA-Spark, Hamamatsu). After deformation in the constriction, the zygotes were pipetted out of the outlet and left to recover for 1 hour in M2 medium at 37°C. The zygotes passed through the microfluidic constriction and their unmanipulated counterparts were transferred to T6 medium in 20-μl droplets covered with mineral oil (M8410; Sigma-Aldrich) (*47*). Zygotes were cultured in a humidified incubator supplemented with 5% CO_2_ at 37°C for 5 days. Developing embryos were scored for survival and embryonic stage from E0.5 to E4.5. A fraction was imaged at each scoring time point.

### Plasmids, in vitro transcription of cRNAs, and oocyte microinjection

As previously described (*11*), we used the following construct pRN3-EzTD-mCherry-VCA. In vitro synthesis of cRNAs was performed using an mMessage mMachine kit (AM1344, Ambion) and subsequent purification with the RNeasy Kit (74106, Qiagen) following the manufacturer’s instructions. cRNAs were centrifuged at 4°C for 45 min at 20,000*g* before microinjection. cRNAs were injected in prophase I arrested oocytes using an Eppendorf Femtojet micro-injector. cRNA translation was allowed for 2 hours before meiosis resumption was triggered.

### Oocyte measurement in the constriction

After deposition in the inlet, the oocyte was driven to the constriction at a moderate flow rate of around 2 μl/min. Once the oocyte reached the constriction, the flow rate was adjusted to 0 μl/min to stop the oocyte in contact with the channel walls without being deformed. The inlet pressure was increased by 0.1 mbar every 2.5 s until the oocyte passed through the constriction. Pressure and flow were recorded every 50 ms for the duration of the oocyte’s passage. Automation and recording were performed using OxyGEN software (Fluidgent). An image of the oocyte was taken every 50 ms on an inverted bright-field microscope (Diaphot 300, Nikon) with a 20× dry objective and a CMOS camera (ORCA-Spark, Hamamatsu). Triggering of image recording and stepwise pressure increase were synchronized using auto-click software (fig. S1B). The contour of the oocytes, excluding the zona pellucida, was obtained using Oocytor plugin (*34*). The contours were then analyzed using Fiji software (*48*) to extract oocyte position and aspect ratio as a function of time since the triggering of stepwise pressure increase and image recording. Because of the experimental variation observed in the *P*_*X*f=0_ measurement between control conditions (fig. S2A), all pressure values were normalized to the median and interquartile range of the *P*_*X*f=0_ measurement for the control condition of each experiment.

### Determination of the equilibrium pressure for a given cell configuration

#### Cell geometry

To find the equilibrium pressure difference ∆P associated with a given cell configuration, we assume that the cell geometry is composed of three different sections (Fig. 3A): (i) a spherical cap on the rear end with a volume $V_{r}=\frac{\pi h_{r}}{6}\left(\frac{3D_{r}^{2}}{4}+h_{r}^{2}\right)$, where $h_{r}$ is the height of cap and $D_{r}$ is the diameter of the channel at the base of the cap; (ii) a partial cone with a volume $V_{\text{cone}}=\frac{\pi }{24\text{tan}\left(\alpha \right)}\left(D_{r}^{3}-D_{f}^{3}\right)$ where $D_{f}$ is the diameter of the channel at smaller base of the partial cone; and (iii) a spherical cap on the front end with a volume $V_{f}=\frac{\pi h_{f}}{6}\left(\frac{3D_{f}^{2}}{4}+h_{f}^{2}\right)$ with $h_{f}$ the height of the cap. The volume of the cell is then calculated as

$$V=V_{f}+V_{\text{cone}}+V_{r}$$ (4)

These volumes can be computed from the channel geometry and the volume conservation condition that require $V=V_{0}$ (where $V_{0}$ is the initial volume of the cell). To finally account for the cortex elasticity (in Eq. 1), we evaluate the total surface area of the cell, composed of two spherical caps and a partial cone, i.e.,

$$A=A_{f}+A_{\text{cone}}+A_{r}$$ (5)

where

$$\begin{array}{c} A_{r}=\pi \left(\frac{D_{r}^{2}}{4}+h_{r}^{2}\right)\\ A_{\text{cone}}=\frac{\pi }{\text{tan}\left(\alpha \right)}\left(\frac{D_{r}^{2}}{4}-\frac{D_{f}^{2}}{4}\right)\;A_{f}=\pi \left(\frac{D_{f}^{2}}{4}+h_{f}^{2}\right) \end{array}$$ (6)

It should be noted that these formulations are valid before the front spherical cap becomes a hemisphere ($P_{Xf=d/2}$). After this stage, the shell will be composed of four geometrical sections, including two spherical caps, a partial cone and a cylinder. We can show however, that at that stage, the motion of the cell is unstable (i.e., cell translation is associated with a drop in pressure difference). Since results presented in this work only relate to the stable branch of the pressure/motion curve, this stage is not considered in our analysis. In the analysis, we lastly assume that the cell is initially a perfect sphere, the initial volume and surface area of the shell are thus $V_{0}=\frac{\pi D_{0}^{3}}{6}$ and $A_{0}=\pi D_{0}^{2}$, where $D_{0}$ is the cell’s initial diameter.

#### Numerical procedure

The solution to the problem relies on finding the relationships between the height and radius of the bases of the caps. Thus, the solution procedure is implemented into two successive stages. (i) In the first stage, the wall of the tapered section of the microchannel is identical to the tangent lines to both spherical caps at the point of contact. With this assumption, the heights of the caps are related to the radii of the bases as follows

$$h_{r}=\frac{D_{r}}{2}\left[\text{tan}\left(\alpha \right)+\sqrt{1+{\text{tan}}^{2}\left(\alpha \right)}\right]$$ (7)

$$h_{f}=\frac{D_{f}}{2}\left[-\text{tan}\left(\alpha \right)+\sqrt{1+{\text{tan}}^{2}\left(\alpha \right)}\right]$$ (8)

This stage lasts until the wall of the tapered section of the microchannel is no longer tangent to the downstream spherical cap. This corresponds to $X_{\text{fcap}}=X_{f}-h_{f}=X_{0}$. (ii) In the second stage, the downstream cap enters the constriction part of microchannel ($D_{R}=d$). Thus, $h_{R}$ is independent from $D_{R}$ and Eq. 8 is no longer valid. However, the tangency condition still holds for the upstream cap, and we again use Eq. 7. Note that in contrast to the experimental procedure, the numerical procedure consists of finding the pressure difference associated with a prescribed cell location therefore avoiding numerical convergence issues related to the problem’s inherent loss of stability.

The numerical procedures for the two aforementioned stages are as follows:

First stage:

1) For a given value of $X_{\text{fcap}}$, we calculate $D_{f}=-2\text{tan}\;\left(\alpha \right)\left(X_{\text{fcap}}-\right.$$\left.X_{0}\right)+d$.

2) We find $h_{f}$ by Eq. 8.

3) Given that $D_{r}=-2\text{tan}\left(\alpha \right)\left(X_{\text{rcap}}-X_{0}\right)+d$ and Eq. 7, we find $X_{\text{rcap}}$ that satisfies the incompressibility condition.

4) We calculate the radii of the spherical caps as $R_{f}=\frac{\frac{D_{f}^{2}}{4}+h_{f}^{2}}{2h_{f}}$ and $R_{r}=\frac{\frac{D_{r}^{2}}{4}+h_{r}^{2}}{2h_{r}}$.

5) We find the surface area A and surface force τ that are calculated by Eqs. 5 and 1, respectively.

6) Aspect ratio is calculated by $\text{AR}=\frac{X_{f}-X_{r}}{2^{*}R_{r}}$.

7) We calculate $P_{r}-P_{f}$ by Eq. 3.

8) We repeat steps (1) through (7) until $X_{\text{fcap}}=X_{0}$

Second stage:

1) We set $D_{f}=d$.

2) For a given value of $h_{f}$, we solve the incompressibility equation to find $X_{\text{rcap}}$. To solve this equation, we again use Eq. 7 and $D_{r}=-2\text{tan}\left(\alpha \right)\left(X_{r}-X_{0}\right)+d$.

3) We calculate the radii of the spherical caps as $R_{f}=\frac{\frac{D_{f}^{2}}{4}+h_{f}^{2}}{2f}$ and $R_{r}=\frac{\frac{D_{r}^{2}}{4}+h_{r}^{2}}{2h_{r}}$.

4) We find the surface area A and surface force τ that are calculated by Eqs. 5 and 1, respectively.

5) Aspect ratio is calculated by $\text{AR}=\frac{X_{f}-X_{r}}{2^{*}R_{r}}$.

6) We calculate $P_{r}-P_{f}$ by Eq. 3.

7) We repeat steps (2) through (6) until $h_{f}=d/2$

The numerical procedures were done on MATLAB software (MathWorks), and the code is available at https://doi.org/10.5281/zenodo.14199390.

### Analytical closed form for the critical pressure

Given the relationships between geometry parameters, we can find an analytical closed form for the critical pressure $P_{c}=P_{Xf=d/2}$. This pressure corresponds to the last iteration of the second-stage simulation procedure. By setting $h_{f}=\frac{d}{2}$ and $D_{f}=d$, and accounting for the incompressibility equation, the diameter $D_{r}^{\left(\text{cr}\right)}$ of the base of the upstream cap is calculated as

$$D_{r}^{\left(\text{cr}\right)}=\text{cosα}\sqrt[{3}]{\frac{\text{sinα}}{{\left(1+\text{sinα}\right)}^{2}}\left[4D_{0}^{3}-\left(2-\text{cotα}\right)d^{3}\right]}$$ (9)

Therefore, the surface area of the shell at $P_{Xf=d/2}$ is calculated as follows

$$A^{\left(\text{cr}\right)}=\frac{\pi d^{2}}{4}\left(2-\text{cotα}\right)+\frac{\pi {\left[D_{r}^{\left(\text{cr}\right)}\right]}^{2}}{4}\left[1+\text{cotα}+\frac{{\left(1+\text{sinα}\right)}^{2}}{{\text{cos}}^{2}\alpha }\right]$$ (10)

At this pressure, the radius of the downstream cap is equal to the constriction radius $R_{f}=\frac{d}{2}$ and the radius of the upstream cap (using trigonometric identities) is calculated as $R_{r}=\frac{D_{r}^{\left(\text{cr}\right)}}{2\text{cosα}}$. Combining these formulas, the close form formula for $P_{Xf=d/2}$ is calculated as

$$P_{c}=4\left\{\tau_{0}+Et_{\text{ch}}\left[\frac{A^{\left(\text{cr}\right)}}{\pi D_{0}^{2}}-1\right]\right\}\left[\frac{1}{d}-\frac{\text{cosα}}{D_{r}^{\left(\text{cr}\right)}}\right]$$ (11)

The above equation can be rewritten as

$$P_{c}=m_{\tau 0}\tau_{0}+m_{E}E$$ (12)

where $m_{\tau 0}=\frac{\partial P_{c}}{\partial \tau_{0}}$ shows the sensitivity of the critical pressure to the surface tension and $m_{E}=\frac{\partial P_{c}}{\partial E}$ measures the sensitivity of the critical pressure to the elasticity of the shell. Comparing Eq. 11 with Eq. 12, we find

$$m_{\tau 0}=4\left[\frac{1}{d}-\frac{\text{cosα}}{D_{r}^{\left(\text{cr}\right)}}\right]\;m_{E}=t_{\text{ch}}\left[\frac{A^{\left(\text{cr}\right)}}{\pi D_{0}^{2}}-1\right]m_{\tau 0}$$ (13)

Thus, Eq. 12 shows that the critical pressure changes linearly with both surface tension and elasticity (Fig. 3, C and D). Furthermore, Eq. 13 can be used to study the effects of determining parameters, such as the constriction diameter d (fig. S3B), the ratio $D_{0}/d$ (fig. S3, G and H), and the inclination angle α (fig. S3C) on $P_{Xf=d/2}$.

### Model correction for a pyramidal geometry instead of the axisymmetric conical geometry

We could consider a truncated or partially pyramidal geometry with a square cross section, the front and rear hemispherical caps remaining unchanged. Given these modifications, the area of the central section (Eq. 5) and the volume of the central section (Eq. 4) must be modified accordingly.

Considering a square cross section for the channel with a side length of $W_{f}=H_{f}=D_{f}$ at the front (intersection of the middle part and the front spherical cap) and $W_{r}=H_{r}=D_{r}$ at the rear of the cell (intersection of the middle part and the rear spherical cap), the values would be modified as

$$V_{\text{pyramid}}=\frac{1}{6\text{tan}\left(\alpha \right)}\left(D_{r}^{3}-D_{f}^{3}\right)$$ (4 bis)

$$A_{\text{pyramid}}=\frac{1}{\text{sin}\left(\alpha \right)}\left(D_{r}^{2}-D_{f}^{2}\right)$$ (6 bis)

### Machine learning approach for mouse oocyte morphological characterization

The machine learning pipeline based on our open-source Fiji plugin Oocytor (*36*) was used as previously described (*37*, *38*) to automatically measure and compare morphological features of control and cVCA oocytes. Oocytor allowed us to automatically segment, extract, and compare 93 morphological features from single images of control and cVCA oocytes before entering the constriction. To evaluate Oocytor’s discriminative performance, we trained and tested a random forest classifier with a fivefold cross-validation scheme (we also tried threefold and 10-fold cross-validation, with little impact on the results). We split the dataset (45 control and 44 cVCA oocytes) in five groups. The random forest classifier was trained on four of five groups, and we evaluated its performance on the fifth group by measuring the accuracy of the classification (percentage of correctly classified oocytes). This was repeated so that each of the five groups would be left out and used for testing once.

### Estimation of the variation in oocyte diameter in mouse and human

Using Oocytor, we reanalyzed the data from (*36*) and (*38*) to measure the diameter of mouse oocytes [4 hours after nuclear envelope breakdown (NEBD)] and human oocytes (at a corresponding stage, 8 hours after NEBD). These timings are identical to those from control and cVCA oocytes. The median diameter for mouse oocytes was 73.09 μm, with a range of 7.5 μm in the 10th to 90th percentiles. The median diameter for human oocytes was 114.7 μm, with a range of 11.3 μm in the 10th to 90th percentiles. Thus, the size range in the 10th to 90th percentiles induces a variation of 0.15 for a *D*_0_/*d* ratio of 1.5 for mouse and human oocytes. Furthermore, the Flinger-Kilinger statistical test showed no significant difference in the variation of the distribution of mouse and human oocyte diameters. The variability of human oocyte size is thus comparable to that of mouse oocytes.

### Meiotic spindle length and chromosomes alignment

Oocytes were incubated 30 min with 0.1 μM SiR-tubulin (SC002, SiR-tubulin Kit, Spirochrome) and Hoechst (5 ng/ml; H6024, Sigma-Aldrich) to label microtubules of the meiotic spindle and DNA of the chromosomes. Images were acquired on a confocal spinning disk microscope (DMI6000B, Leica and CSU-X1 Spinning disk, Yokogawa) enclosed in a thermostatic chamber (Life Imaging Service) using a Plan-APO 40×/1.25–numerical aperture objective and a charge-coupled device camera (Retiga 3, QImaging). Metaphase plate width and meiotic spindle length were measured by manually placing bounding boxes on DNA fluorescent signal and microtubule fluorescent signal respectively using the Fiji software (National Institutes of Health). Measurements were done only on spindles parallel to the imaging plane (*21*).

### Statistical analysis

Appropriate statistical test was applied according to data normality (determined by Shapiro-Wilk and Kolmogorov-Smirnov tests) and content (paired observations and/or equal variance). Respective tests and *n* numbers are indicated in the figure legends. Statistical analysis was performed using GraphPad software (PRISM).
